# Effect of high pressure on hydrocarbon-degrading bacteria

**DOI:** 10.1186/s13568-014-0077-0

**Published:** 2014-10-15

**Authors:** Martina Schedler, Robert Hiessl, Ana Gabriela Valladares Juárez, Giselher Gust, Rudolf Müller

**Affiliations:** 1 Institute of Technical Biocatalysis, Hamburg University of Technology, Hamburg 21073, Germany; 2 Institute for Product Development and Mechanical Engineering Design, Hamburg University of Technology, Hamburg 21073, Germany

**Keywords:** Biodegradation, High pressure, Hydrocarbons, Naphthalene, n-Hexadecane

## Abstract

The blowout of the Deepwater Horizon in the Gulf of Mexico in 2010 occurred at a depth of 1500 m, corresponding to a hydrostatic pressure of 15 MPa. Up to now, knowledge about the impact of high pressure on oil-degrading bacteria has been scarce. To investigate how the biodegradation of crude oil and its components is influenced by high pressures, like those in deep-sea environments, hydrocarbon degradation and growth of two model strains were studied in high-pressure reactors. The alkane-degrading strain *Rhodococcus qingshengii* TUHH-12 grew well on n-hexadecane at 15 MPa at a rate of 0.16 h^−1^, although slightly slower than at ambient pressure (0.36 h^−1^). In contrast, the growth of the aromatic hydrocarbon degrading strain *Sphingobium yanoikuyae* B1 was highly affected by elevated pressures. Pressures of up to 8.8 MPa had little effect on growth of this strain. However, above this pressure growth decreased and at 12 MPa or more no more growth was observed. Nevertheless, *S. yanoikuyae* continued to convert naphthalene at pressure >12 MPa, although at a lower rate than at 0.1 MPa. This suggests that certain metabolic functions of this bacterium were inhibited by pressure to a greater extent than the enzymes responsible for naphthalene degradation. These results show that high pressure has a strong influence on the biodegradation of crude oil components and that, contrary to previous assumptions, the role of pressure cannot be discounted when estimating the biodegradation and ultimate fate of deep-sea oil releases such as the Deepwater Horizon event.

## 1 Introduction

From April to July 2010, 779 million litres of oil were released into the Gulf of Mexico when the Deepwater Horizon (DWH) drilling rig platform exploded (Atlas and Hazen [2011]). This event was the largest marine oil spill in history. However, there are other anthropogenic and natural sources of oil released into the oceans. The National Research Council ([2003]) estimated an overall input of about 1.3 Mt oil per year into the marine environment from all sources. Approximately 47% originates from natural seeps and the remaining 53% comes from activities related to the extraction, transportation and consumption of crude oil or refined products (National Research Council [2003]).

In case of the Deepwater Horizon accident, it is estimated that a substantial proportion of the hydrocarbons entering deep plumes was converted to biomass (about 0.8–2 · 10^10^ mol carbon) (Shiller and Joung [2012]). Substantial bacterial blooms were observed in deep waters in the months following the blowout, indicating that indigenous hydrocarbon-degrading bacteria were enriched by the released crude oil and methane (Bælum *et al.*[2012]; Hazen *et al.*[2010]; Kessler *et al.*[2011]; Redmond and Valentine [2012]; Valentine *et al.*[2010], [2012]). Oil-degrading bacteria have evolved over millions of years and are ubiquitous in the marine environment. Up to now, more than 200 bacterial, algal and fungal genera, representing over 500 species, are described as capable of hydrocarbon degradation (Yakimov *et al.*[2007]). Therefore, natural bacterial activity is an important mechanism for environmental remediation of oil spills. Much research has been done on crude oil biodegradation in the marine environment (e.g. Colwell *et al.*[1977]; Head *et al*. [2006]; Leahy and Colwell [1990]; Powell *et al*. [2004]; Yakimov *et al.*[2007]), especially in the context of the DWH blowout (Hazen *et al*. [2010]; Kessler *et al.*[2011]; Redmond and Valentine [2012]; Valentine *et al*. [2012]).

The DWH drilling rig well, from which oil and gas flowed out uncontrollably for three months, was located 1,500 m below the sea surface. Such deep-sea environments are characterized by extreme conditions. These include low temperatures of 3°C (±1°C) (Jannasch and Taylor [1984]) and high hydrostatic pressures up to 110 MPa in the Mariana Trench, the deepest site existing in the ocean at 10,994 m (±40 m) (Abe and Horikoshi [2001]; Gardner and Armstrong [2011]). However, only a limited number of studies regarding the capabilities of bacteria to degrade oil and hydrocarbons have been conducted under high pressure (Grossi *et al*. [2010]). Despite the detection of pressure-induced differences in growth and hydrocarbon utilisation (Schwarz *et al.*[1974], [1975]), most reports have investigated oil biodegradation only at surface pressure (0.1 MPa) (Cui *et al*. [2008]; Tapilatu *et al.*[2010]; Wang *et al.*[2008]), and corresponding results may not be applicable to the deep sea. Thus, oil biodegradation processes under extreme deep-water conditions are not well understood. Although the rate and extent of hydrocarbon degradation at elevated pressures has been understudied, an understanding of the impact of elevated pressures on biodegradation is increasingly critical in the wake of expanding drilling in deep waters.

This study aims to improve our understanding of microbial degradation processes of crude oil at in situ deep-sea conditions. In particular, we consider the effects of high pressure on hydrocarbon biodegradation using high-pressure lab technology. Oil is one of the most complex mixtures of organic compounds known, containing more than 17,000 distinct components (Hassanshahian and Cappello [2013]; Head *et al.*[2006]). To simplify our approach, we investigated the biodegradation of two representatives of the main fractions of oil by two different bacterial model strains: *Rhodococcus qingshengii* TUHH-12, a degrader of the alkane n-hexadecane; and *Sphingobium yanoikuyae* B1, a bacterium capable of utilizing naphthalene, a polycyclic aromatic hydrocarbon (PAH) (Gibson *et al*. [1973]). *R. qingshengii* and *S. yanoikuyae,* as well as other species of the genera *Rhodococcus* and *Sphingobium* have been isolated from deep-sea sediments (Colquhoun *et al.*[1998a]; Cui *et al.*[2008]; Heald *et al*. [2001]; Peng *et al.*[2008]; Tapilatu *et al*. [2010]; European Nucleotide Archive [2014]; Wang and Gu [2006]). Recently, *Rhodococcus* sp. and *Sphingobium* sp. were found to be present in sediment samples collected in May 2011, about 2 and 6 km away from the wellhead of the DWH (Liu and Liu [2013]). In our experiments, both strains *R. qingshengii* TUHH-12 and *S. yanoikuyae* B1 were incubated at 0.1 MPa and at increasing pressures to determine the influence of pressure on the growth and hydrocarbon-degradation abilities of these strains.

## 2 Materials and methods

### 2.1 Microorganisms

The alkane-degrading bacterium *R. qingshengii* TUHH-12 [DSM No.: 46766] was isolated from a seawater sample located directly beneath an ice cap swimming on the water during an expedition to the island of Spitzbergen, Norway, by Prof. Hauke Trinks (Hamburg University of Technology). The genome of this strain was recently sequenced (Lincoln SA, Penn State University, unpublished data).

*S. yanoikuyae* B1, purchased from DSMZ [DSM No.: 6900], was originally isolated from a polluted stream by Gibson *et al*. [1973]. This bacterium was preliminary identified as *Beijerinckia* species, but has later been reclassified as *Sphingobium yanoikuyae* B1 (Khan *et al.*[1996]). This strain is known to degrade different aromatic and polycyclic aromatic hydrocarbons (Gibson *et al.*[1973]; Lang [1996]).

### 2.2 Cultivation conditions

*R. qingshengii* TUHH-12 was cultivated on minimal-mineral medium with n-hexadecane as the sole carbon source. The medium consisted of 2.6 g Na_2_HPO_4_, 1.33 g KH_2_PO_4_, 1 g (NH_4_)_2_SO_4_ and 0.20 g MgSO_4_ · 7 H_2_O dissolved in 1000 mL of demineralized water. The medium was adjusted to pH 7. After sterilisation, 5 mL of trace element solution and 1 mL of vitamin solution were added. Both solutions were prepared as described in DSMZ methanogenium medium 141 and autoclaved or sterile filtered separately ([DSMZ 2012]). Cultures were incubated at room temperature and mixed at 200 rpm. The strain was kept on agar plates containing the same medium with 15 g/L agar added for solidification.

*S. yanoikuyae* B1 was cultured in Brunner mineral medium or on agar plates according to the DSMZ medium 457 (DSMZ [2012b]) at 30°C or at room temperature and 200 rpm. Naphthalene was used as sole carbon source.

### 2.3 Biodegradation experiments at high pressure

**Figure 1:**
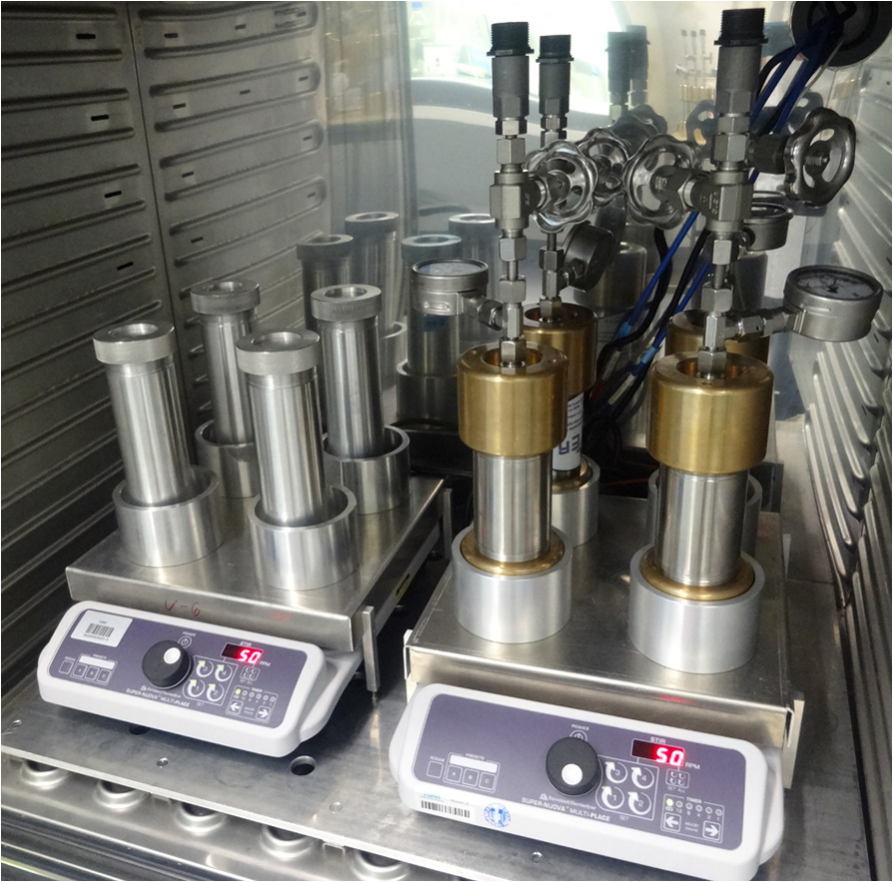
**High-pressure reactors and control reactors.** High-pressure reactors (right, made from stainless steel and bronze, max. pressure 40 MPa, pressurizing with N_2_ gas, 160 mL volume) and aluminum control reactors (left, max. 0.1 MPa, 160 mL volume) were used for cultivation of hydrocarbon degraders at high and ambient pressure. The cultures were mixed with magnetic stirrers.

Ten high-pressure reactors consisting of stainless steel cylinders capped with bronze lids were used to simulate and to investigate biodegradation under elevated pressures as they occur in deep-sea environments. Additionally, ten aluminium reactors with the same geometry were used, serving as controls to monitor biodegradation under atmospheric pressure in simultaneous experiments (Figure 1). Both reactor types had a volume of 160 mL. For experiments with *R. qingshengii* TUHH-12, 20 mL mineral medium was filled into sterilized glass vials and supplemented with 1 mM n-hexadecane. For cultivation of *S. yanoikuyae* B1, 20 mL Brunner medium and 1.77 mM naphthalene were used. The amount of carbon in 1.77 mM naphthalene is equal to the amount of carbon in 1 mM n-hexadecane. The media were inoculated with a grown preculture of the respective bacterial strain, constituting 10% of the total volume. The vials were placed inside the reactors. The high-pressure reactors were pressurized with nitrogen gas up to 15 MPa (equivalent to 1,500 m DWH well depth). The cultures were incubated at room temperature. Since the oil components used in these experiments are nearly insoluble in water, stirring rates affect biodegradation rates; therefore, efficient mixing of the cultures was ensured by magnetic stirring at 200 rpm. Due to the immiscible two-phase system of oil and water and the impracticality of subsampling at high pressure, no representative samples could be taken from the reactors to monitor oil concentrations. Thus, for each point in time in a diagram the content of one reactor was processed. Before opening a reactor containing n-hexadecane, it was cooled for 5 h at 4°C to minimise evaporation of n-hexadecane. Bacterial growth was measured and the hydrocarbon concentrations were analysed to quantify the degree of biodegradation. In several repetitions of the experiments the effects of pressure were the same. Only slightly different growth and degradation rates were observed due to different sampling times and slightly differing inoculation cell numbers. Thus, the diagrams presented represent the typical course of growth and hydrocarbon degradation.

### 2.4 Determination of growth of *S. yanoikuyae* B1 with glucose at ambient and high pressure

In order to determine, whether the growth of *S. yanoikuyae* B1 was inhibited when growing on a non-toxic substrate at high pressure, we incubated the bacteria in Brunner medium with 1% glucose (w/v) at room temperature. After incubation for 44.5 hours at 0.1 MPa and at 15 MPa, the reactors were opened and cell numbers were determined.

### 2.5 Bacterial growth

Colony forming units (CFUs) of *R. qingshengii* TUHH-12 were determined by spreading 5 μL of the culture on Luria-Bertani (LB) agar plates in triplicate. The colonies were counted after 3 to 4 days of incubation at room temperature.

For *S. yanoikuyae* B1, plate counts were performed with R2A agar medium (DSMZ medium 830 (DSMZ [2012c])). The colonies were counted after 2 to 3 days of incubation at room temperature.

### 2.6 Quantification of the biodegradation of hydrocarbons by gas chromatography–mass spectrometry

After growth of the cultures, the remaining n-hexadecane or naphthalene was extracted from the complete culture medium of each reactor with 5 mL of n-hexane. Dodecane or n-hexadecane were added before extraction as internal standard respectively. An aliquot of 1 μL of the apolar phase, containing the hydrocarbon, was injected with a split ratio of 28:1 into a Hewlett-Packard 5890 Series II gas chromatograph (GC) coupled to a Hewlett-Packard 5971A mass selective detector. The GC was equipped with an Agilent HP-5MS column (30 m length, 0.25 mm internal diameter) and helium was used as carrier gas. The injector temperature for both n-hexadecane and naphthalene was increased from 80°C to 200°C at a rate of 0.5°C/s. The oven temperature program was as follows: an initial temperature of 80°C was increased to a final temperature of 200°C at a rate of 15°C/min, with a final 1 min hold at 200°C. The mass spectrometer was operated in full scan mode over 50–650 amu. The MS transfer line temperature was held at 320°C and the ion source temperature at 180°C.

### 2.7 Detection of hydroxylated intermediates in naphthalene conversion

For detection of hydroxylated intermediates in naphthalene conversion, the colourimetric method described by Arnow ([1937]) was used. After 3 min centrifugation of 1 mL of a grown culture at 13,000 rpm, 200 μL of the supernatant were supplemented by 200 μL of the following reagents, in the order given, mixing well after each addition: 0.5 N HCl, nitrite-molybdate reagent, 1 N NaOH. If catechol or 1,2-dihydroxynaphthalene were present, a yellow colour resulted after addition of HCl and nitrite-molybdate reagent, and a red colour appeared after addition of NaOH. In the case of monohydroxylated compounds like salicylate or monohydroxynaphthalene the solution remained yellow.

## 3 Results

### 3.1 Degradation of n-hexadecane by *R. qingshengii* TUHH-12 at ambient and high pressure

**Figure 2:**
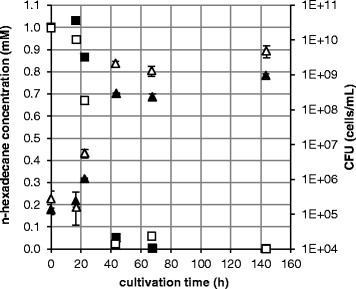
**Degradation of n-hexadecane at 0.1 MPa vs. 15 MPa by*R. qingshengii*TUHH-12.** The CFUs were determined by plate counting and n-hexadecane concentrations were measured by gas chromatography–mass spectrometry. CFUs were determined in triplicate and standard deviations are shown. △ CFU at 0.1 MPa, **▲** CFU at 15 MPa, □ n-hexadecane concentration at 0.1 MPa, ■ n-hexadecane concentration at 15 MPa.

*R. qingshengii* TUHH-12 was cultivated on n-hexadecane as the sole source of carbon and energy. *R. qingshengii* TUHH-12 was found to grow well and to mineralize this hydrocarbon at atmospheric pressure (0.1 MPa) as well as at high pressure (15 MPa). At 15 MPa the degradation and growth behaviour was slightly different from that at atmospheric pressure (Figure 2). In both cases, a lag phase of 16 to 17 h was followed by an exponential growth phase and a stationary phase starting after 43 to 44 h of incubation. However, the growth rate of *R. qingshengii* TUHH-12 in the exponential phase was 0.36 h^−1^ at ambient pressure, from 17 h to 43 h, compared to 0.16 h^−1^ at high pressure, from 16 h to 44 h. In the stationary phase a higher cell density was reached at 0.1 MPa than at 15 MPa. The rate of degradation of n-hexadecane was 0.035 mM/h at ambient pressure from 17 to 43 h, and 0.019 mM/h at high pressure, from 16 to 44 h. In control experiments without bacteria at 15 MPa the n-hexadecane also slowly decreased although with a much slower rate of 0.007 mM/h.

### 3.2 Degradation of naphthalene by *S. yanoikuyae* B1 at ambient and high pressure

**Figure 3:**
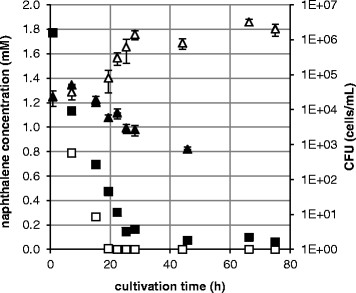
**Growth of*S. yanoikuyae*B1 on naphthalene at 0.1 MPa vs. 13.9 MPa.***S. yanoikuyae* B1 was cultivated at room temperature. CFUs were determined in triplicate and standard deviations are shown. △ CFU at 0.1 MPa, **▲** CFU at 13.9 MPa, □ concentration of naphthalene at 0.1 MPa, ■ concentration of naphthalene at 13.9 MPa.

*S. yanoikuyae* B1 was incubated on naphthalene at high pressure (13.9 MPa) and at atmospheric pressure. The growth of *S. yanoikuyae* B1 on this PAH was strongly inhibited by high pressure. Bacteria grew at 0.1 MPa with a lag phase of 15 h, an exponential phase with a growth rate of 0.33 h^−1^ (from 15 to 28 h) and reached stationary phase at 28 h of incubation (Figure 3). At 13.9 MPa, however, CFUs of *S. yanoikuyae* B1 decreased after 15 h cultivation time until no CFUs could be counted after 66 h.

In 0.1 MPa experiments, the analysis of remaining naphthalene in the medium showed that the substrate was degraded completely after 19 h. The degradation rate of naphthalene, from 7 h to 19 h, was 0.064 mM/h. Because the bacteria did not grow at 13.9 MPa, we expected that at this pressure no naphthalene would be degraded at all. However, we also observed a decrease in substrate concentration at this pressure. With a conversion rate of 0.054 mM/h (from 7 h to 25 h), 96.6% of the naphthalene was converted after 75 h of incubation. After 66 h of incubation at elevated pressure, the initially colourless culture medium turned brown, while at ambient pressure the culture showed no change of colour.

In control experiments without bacteria about 20.8% of the initial naphthalene was found to be lost after 19 days of incubation at 14.2 MPa by evaporation of the highly volatile naphthalene. With a loss of about 25.3% at 0.1 MPa there was no significant difference to the incubation at high pressure.

**Figure 4:**
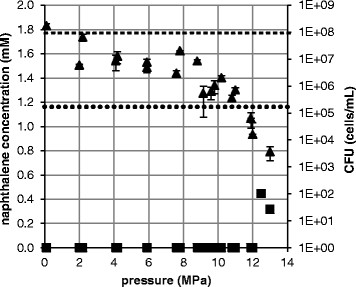
**CFU counts (▲) of*S. yanoikuyae*B1 growing on naphthalene (■) at different pressures.***S. yanoikuyae* B1 was cultivated at room temperature. The CFUs were counted after an incubation time of 70 h and determined in triplicate. Standard deviations are shown. The dashed line indicates the starting naphthalene concentration (▬ ▬ ▬), the stippled line is the starting cell number (● ● ●) at 0 h.

Since *S. yanoikuyae* B1 did not grow at 13.9 MPa, we conducted additional experiments to determine the maximum pressure at which growth was possible. *S. yanoikuyae* B1 was incubated for 70 h on naphthalene at different pressures in the range between 0.1 MPa and 13 MPa (Figure 4). In the range of 0.1 to 8.8 MPa, the CFU counts remained relatively constant, but decreased when 8.8 MPa was exceeded. In incubations at and above 12 MPa no growth occurred after 70 h cultivation time and viable cell counts were lower than at the start of the incubation.

The naphthalene concentration decreased to below the limit of detection under both ambient pressure and pressures up to 12 MPa. At 12.5 MPa and 13 MPa, 25.2% and 17.9% of the original naphthalene remained, respectively, although CFUs did not increase. While at 0.1 MPa no change of colour could be observed, at 12.5 MPa, after 70 h of incubation, the medium had turned brown. In the test with the colour reagent of Arnow ([1937]), we found no colour, indicating that neither mono- nor dihydroxylated compounds like 1,2-dihydroxynaphthalene, catechol or salicylate were formed. We therefore assume that the formation of the brown colour was due to the polymerization of either quinones or aromatic ring cleavage products.

### 3.3 Growth of *S. yanoikuyae* B1 with glucose at different pressures

While *S. yanoikuyae* grew well on glucose at 0.1 MPa, at 15 MPa no growth at all was observed. After 44.5 hours the cell number had decreased from 5.98 · 10^5^ to 1.7 · 10^5^ cells per mL at 15 MPa. These results indicate that at high pressure it is not the conversion of naphthalene or its metabolites but rather another central function in *S. yanoikuyae* B1, which is inhibited.

## 4 Discussion

### 4.1 Degradation of n-hexadecane by *R. qingshengii* TUHH-12 at ambient and high pressure

*R. qingshengii* TUHH-12 degraded the alkanes n-hexadecane, decane and tetracosane. *R. qingshengii* has been found to assimilate and mineralize different hydrocarbons including benzene, toluene, xylenes, naphthalene, n-dodecane (Benedek *et al*. [2013]). However, until now, nothing was known about degradation capabilities of *R. qingshengii* at other than atmospheric pressure. Our experiments showed that the growth rate of *R. qingshengii* TUHH-12 at ambient pressure (0.36 h^−1^) was slightly higher than at 15 MPa (0.16 h^−1^). This leads to the conclusion that a pressure of 15 MPa has a slightly negative effect on the growth of this bacterium, suggesting it can be classified as a piezotolerant organism. These findings are confirmed by the work of Colquhoun *et al*. ([1998b]) and Heald *et al*. ([2001]), who showed that certain *Rhodococcus* strains were able to grow at even higher pressures of 40 MPa and 60 MPa on glucose yeast extract medium.

### 4.2 Effects of elevated pressure on the naphthalene degradation by *S. yanoikuyae* B1

The model strain *S. yanoikuyae* B1, used for PAH degradation in our high-pressure experiments, is capable of utilising a variety of aromatic compounds including biphenyl, anthracene, phenanthrene and naphthalene (Gibson *et al*. [1973]), as well as toluene, cyclohexane and 1,3,5-trimethylbenzene (Lang [1996]) as carbon sources for growth. We found significant differences in growth of *S. yanoikuyae* B1 with naphthalene at different pressures. These effects occurred at pressures lower than those typically assumed to be the threshold for pressure effects. First significant effects of high pressure on cellular components and processes of bacteria were found to start at 20 MPa, affecting the RNA transcription (Yayanos and Pollard [1969]). Modifications of membrane fluidity were shown to occur at pressures above 100 MPa (Hauben *et al.*[1997]) and protein denaturation was observed at more than 400 MPa (Aertsen *et al.*[2009]).

In our experiments at 0.1 MPa *S. yanoikuyae* B1 was able to grow with naphthalene, whereas at 13.5 MPa bacteria did not grow at all and after 66 h of incubation, cells were no longer viable. A similar behaviour was found with glucose as carbon source. Thus, we conclude that *S. yanoikuyae* B1 is a piezosensitive strain that grew best and utilized naphthalene at an optimal rate at ambient pressure. Nevertheless, *S. yanoikuyae* B1 still metabolized naphthalene at 13.9 MPa, although slower than at 0.1 MPa, so that after 75 h of incubation 96.6% of the substrate was converted. We need to emphasize that the naphthalene-degradation capability of *S. yanoikuyae* B1 is much less sensitive to high pressure than growth is. This indicates a new type of piezosensitivity, which, to our knowledge, was not described in literature before.

We can only speculate about the reasons for the observed changes in growth and degradation ability of *S. yanoikuyae* B1 at high pressure. Even a combination of several pressure-induced effects is possible. The investigation of the specific reasons for the strong inhibition of growth and rather slight inhibition of naphthalene conversion of *S. yanoikuyae* B1 by high pressure emerges from our study as a new research topic.

So far in predicting the behavior and degradation of oil spills in deep-sea environments all models use data obtained at ambient pressure for calculating degradation rates. The results presented here show, that the effect of pressure cannot be neglected. When our data obtained under high pressure were included in a model describing the fate of the oil in the case of the Deepwater Horizon, the model predicted the observed changes in oil concentrations much better (Lindo-Atichati *et al.*[2014]).

Our experiments show that pressure affects both bacterial growth and hydrocarbon-degrading activity at pressures much lower than previously reported in the literature as they occur at modern deep-sea drilling sites. Consequently, pressure effects need to be considered as a crucial factor in predictions of oil biodegradation in deep waters.

## Competing interests

The authors declare that they have no competing interests.

## Authors’ contributions

MS participated in the design and performance of the experiments and drafted the manuscript. RH carried out the experiments and analysed the data. AGVJ participated in the design and coordination of the experiment, isolated the strain *R. qingshengii* TUHH-12, and contributed in the drafting of the manuscript. GG participated in the experimental design and contributed in the drafting of the manuscript. RM conceived of the study, participated in its design and contributed in the drafting of the manuscript. All authors read and approved the final manuscript.
